# Illness and Treatment Perceptions of Patients with Epilepsy Attending Treatment at a Tertiary Hospital in Addis Ababa – A Qualitative Exploration

**DOI:** 10.4314/ejhs.v31i3.15

**Published:** 2021-05

**Authors:** Bezawit N Demissie, Abenet T Mengesha, Bruck M Habte

**Affiliations:** 1 School of Pharmacy, College of Health Sciences, Addis Ababa University, Ethiopia; 2 School of Medicine, College of Health Sciences, Addis Ababa University, Ethiopia

**Keywords:** Patient perceptions, Kleinman's model, Necessity-Concerns framework, Epilepsy, Qualitative research, Ethiopia

## Abstract

**Background:**

Epilepsy can be a large economic burden in countries where appropriate treatment is not taken due to religious and psychosocial beliefs. The objective of this study was to explore the perceptions and experiences of patients with epilepsy attending their treatment at Tikur Anbessa Specialized Hospital about their illness and treatment.

**Methods:**

A qualitative exploratory design with an in-depth interview was followed. Fourteen purposively selected patients were used until saturation of key emerging themes was achieved.

**Results:**

The finding showed that study participants expressed perceptions towards their illness including about its cause, timeline, severity and psychosocial consequences which at times may be considered different from the biomedical version. They also expressed concerns about their treatment, reported about social and psychological issues and in certain cases dissatisfaction with their healthcare providers. Such issues may have led to delays in treatment-seeking and non-adherence to recommended treatment regimens and as well use of traditional medicine and spiritual healing. On the other hand, reasons that were reported to positively influence their treatment experiences include necessity perceptions about their medications, family support and use of spiritual healing alongside their biomedical treatment.

**Conclusion:**

The healthcare provision should improve to cater to these groups of patients including instituting chronic care management and appropriate health education provision.

## Introduction

Epilepsy is one of the most common neurological diseases globally and has its own consequences on the physical, economic and social life of the patients (1). About 50 million people worldwide have epilepsy, reaching 1% of the general population with 80% of them living in the developing world (2). The prevalence of epilepsy in the central part of Ethiopia was also reported to be 5.2/1000 population as reported in a large community based epidemiological study (3). Patients related factors such as disbelief of the diagnosis, refusal to take medication and uncertainty about the necessity for drugs were some of the factors affecting adherence to treatment for epilepsy (4). Poor treatment outcome among epileptic patients is related to the level of adherence (5). Exploring patients' illness perception is an important factor to predict patients' adherence behavior (6).

In-depth understanding of patient opinion and judgment on their medication could create communication among health professionals and patients themselves which could result in a better treatment outcome as has been demonstrated for other conditions (7). The purpose of this paper is to contribute to the understanding of the perception of patients with epilepsy and their treatment and the possible reasons for patient's non-adherence to antiepileptic medications.

## Methods

**Study setting:** The study was conducted in Tikur Anbessa Specialized Hospital, Addis Ababa, Ethiopia. The hospital has a Neurology Clinic that serves at least 12,000 ambulatory patients annually. The clinic was run at the time of the study by two consultant neurologists, 10 to 12 residents in the Neurology and Internal Medicine Specialty programs and five nurses.

**Study approach:** A qualitative exploratory approach was used to gain patient insights into the perceptions and experiences regarding epilepsy and its treatment.

Participants and recruitment: Patients with epilepsy who are following treatment in the outpatient specialty clinic of the hospital were selected using a purposive sampling procedure. The criteria for inclusion of study participants included being 18 years of age or above, on anti-epileptic medications for at least a year and not having any other serious debilitating co-morbidity or overt psychiatric condition and able to communicate in Amharic, the official language of the country that is also widely spoken. Being a healthcare provider was an exclusion criterion. Additionally, efforts were made to ensure variation in age, sex, education, duration of illness, type of epilepsy and among other factors.

Study participants were recruited with the support of the Clinic nurses who provided information about the study to the patients and then helped with the preliminary identification of patients for possible inclusion in the study. Following this, the first author who identified herself as a student researcher approached patients for further screening to check eligibility. Those who fulfilled the criteria were then provided further information about the study and their consent sought for inclusion in the study. Accordingly, 14 patients were recruited as study participants based on the principle of data saturation. Their number was determined based on saturation of interview themes which was achieved at participant number 11 but three more patients were interviewed after the saturation of data was attended to determine real saturation was reached. Data collection: Data collection was carried out by the first author, a female researcher who has a pharmacy background with some training on qualitative research methods. Data collection was made using in-depth interview while field notes were taken of the interview settings, study participants' nonverbal message and any other issue that was relevant. The interviews ranged from 20 to 30 minutes and were carried out in a secluded place of the hospital compound which had minimal noise and disturbance. The interviews, which were audio-recorded, were carried out in Amharic which is the official language and widely spoken in Addis Ababa. The interview guide, which was an adaptation of Kleinman, (1978), consisted of open-ended questions related to patients' perception of epilepsy and its treatment (Annex).

**Data analysis:** The audio-recorded data were transcribed verbatim into Microsoft (MS) Word and then analyzed along with field note using the thematic approach. The first author carried out data analysis using color coding in collaboration with the last author, a social pharmacy researcher with advanced training in qualitative research methods, soon after the first three interviews to guide further data collection until saturation of themes achieved. The analysis was informed by Kleinman's, (1978) model and Horne's medication Necessity-Concerns conceptual framework (Horne et al., 2007). This did not, however, limit the open coding of other data which did not fit with the above models. Data was organized or categorized into themes and subthemes. At this stage, the second author, a clinician who specialized in neurology and regularly interacted with patients with epilepsy was involved in the analysis and interpretation. Finally, synthesis and selected participant quotes were translated from Amharic into English and reported.

**Trustworthiness:** The trustworthiness was enhanced by applying the following measures, most of which adhere to recommendations made by the consolidated criteria for reporting qualitative studies (10). An effort was made to recruit patients that have a wide variety in terms of socio-demographic characteristics, year of onset of epilepsy and type of epilepsy. The investigators took detailed field notes, used an audio recorder with the consent of the study participants and presented a summary of the major findings to selected study participants to enhance credibility of the study. Furthermore, the analysis and interpretation were conducted collaboratively among the three authors who had different backgrounds and allowed for different perspectives and thus enhanced the reliability of the findings.

**Ethical Consideration:** This study was approved by the Ethics Committee of the School of Pharmacy, Addis Ababa University (ERB/SOP/61/04/2016). A formal letter was also written to the Department of Neurology from the Department of Pharmaceutics and Social Pharmacy, SOP/CHS/AAU, and permission was obtained to conduct the study. Permission was also sought and obtained from clinical staff working at the clinic where patients were recruited. An informed consent process was followed during participant recruitment while privacy, confidentiality, and anonymity were maintained throughout the study conduct and report writing.

## Results

**Participants' characteristics:** There were fourteen study participants who had a median age of 34 years. Among the participants, eight were males, ten were followers of Orthodox Christianity and eight had postsecondary education (Table 1). The mean number of years of living with epilepsy for males was 14 years and 16 years for females. All participants were on seizure medication, the most common of which were phenobarbital, carbamazepine, and phenytoin.

**Table 1:** Socio-demographic characteristics of study participants (n=14)

Socio-Demographic Characteristics		Number
Age	18–30	4
	31–40	4
	41–50	2
	51–60	4
Sex	Female	6
	Male	8
Religion	Orthodox	10
	Protestant	1
	Muslim	3
Marital status	Single	6
	Married	8
Education	Primary	3
	Secondary	3
	University/college	8
Monthly	<1000	2
income (Birr)	1001–2000	7
	>2000	5

The findings of the study revealed that different terms were used to describe epilepsy in Amharic language. These include “*Azurit*” and “*Yemitel Besheta*” which literally means something that makes them fall. Five key themes emerged from the data: (i) perception about the illness (its cause, symptoms, timeline, severity and consequence); (ii) perception about the treatment (its necessity beliefs and concerns about safety, availability and affordability towards medications; and towards traditional medicines); (iii) relationships with health care providers; (iv) family support; and (v) coping procedures (such as delay in treatment seeking and medication taking behaviors and lifestyle coping mechanisms).

***Perception about the illness***

1. **Cause**: Study participants cited different causes for their epilepsy including the evil spirit, heredity, starvation, fever when they were a child and problem in the brain. Furthermore, they stated different lifestyles related activities such as cigarette smoking, alcohol drinking and *khat* chewing and stress, frustration, as exacerbating factors for their epilepsy.
*“It is caused by starvation. Stress, going outside at night time and also if you fall while you are a child it can change to epilepsy.” (P1, male)*
*“There is something in my family … I relate it with evil spirit. My grandmother worships idol and I wonder if that caused it. Another thing that I think is … may be … when I got born they didn't guard me from bad spirits. You know there are things where your family should protect you from you like ‘lekefet’ were you got exposed to bad spirits.” (P2, male)*
2. **Symptoms:** Most study participants mentioned symptoms were falling, absences (behavioral and mental arrest for few seconds), biting of the tongue and chewing, ‘feeling hot’, rapid blinking of the eyelids, excessive salivation and unconsciousness.
*“First it gives you a sign like feeling hot … and I feel when my blood rises.” (P3, male)*
*“Almost twice, three times… I found myself in another person house. Without knowing it I walk myself to someone else's home. Once I found myself near to the traffic road.” (P4, male)*
3. **Timeline:** Relative to those participants who had epilepsy from childhood, those who had it at an adult age expect to get cured. Some patients believe that the illness is lifelong but they take the medications to control their symptoms. Study participants who think their illness is caused by trauma mentioned that the illness takes longer onset from the time they experience the trauma. On the other hand, participants who described their illness as caused by evil spirits and feeling frightened reported that the illness has fast onset.
  *“My family told me when I was a kid I saw a very scary movie that night I got frightened, disturbed and got sick right away.”* (*P3, male*)
4. **Severity:** All most all of the study participants described epilepsy as a very severe disease. They describe it as severe especially since ‘no one’ understands what they are experiencing.
  *“I have never seen such a difficult disease; no one will understand you because other times you look healthy. This disease doesn't kill you rather it makes your life partial.”* (*P5, male*)
*“For me, it is a very severe disease because… Saying to someone you have kidney or heart disease is very easy relative to saying you have epilepsy.” (P6, female)*
5. **Consequences:** Study participants have reported about different emotional, social and economic consequences that they have encountered in relation to their illness. In relation to the emotional consequence, most reported experiencing depressed mode. They mentioned hating themselves and isolation from other people because of the fear of getting emotionally hurt. They mentioned that the illness makes them not to express themselves. Some respondents mentioned that they feel inferior compared to other healthy individuals.
  *“…You hate things out of nowhere and also you hate being with other people. Even you lose your appetite I don't know if that is related to the disease or not. These feelings can happen before or after the symptom… But the depression can last for a week.”* (*P2, male*)
  *“Whether you believe or not I don't have a girlfriend till now… I think the disease made me not to express myself…I got scared of telling people about it. I got worried…What if they don't accept me as I am?”* (*P3, male*)
  Some also expressed that the illness negatively affected their livelihoods such as jobs and education.
*“I do technical works in my profession… Mostly I drove a car when I moved from one place to another but nowadays I am stopping that because I am scared of the seizure while driving a car.” (P7, male)*
*“I stopped my education when I was a child because I experience a seizure in a school. I wanted to continue my education but I couldn't… Due to frequent attacks of seizure.” (P4, female)*
  Some study participants have experienced stigma from society and express fear and would not be comfortable to reveal about their illness, be it in the workplace or at school. They expressed societal views such as not thinking they are fit for the work as any other individual and that the disease is transmittable. They hate being called sick and peoples gossiping about their illness.
*“The society has a bad impression about it. Even people who are assumed to be educated see you as inferior. They think you cannot perform or work as others do.” (P6, female)*
*“I know this illness is very abominable in the society and also scary. I know people are scared of a person with epilepsy because they think it can be transmitted through physical contact… so basically it makes you to be detached and uncommunicative. Because I know this… I was terrified when I heard that I have epilepsy.” (P7, male)*

***Perception about the treatment***

1. **The necessity of the medication: Study:** participants believe that modern medicines are appropriate for their conditions but with different reasons. They compare the time before they start the medication to see their improvement.
  *“The medications are very important. It decreases the frequency of the seizure and it makes you not to worry. I wouldn't be here talking to you if I wasn't taking the medications; now I have confidence in the medications.”* (*P2, male*)
2. **Safety concerns related to the medications:** Some patients reported experience of side effects and some are worried because they are taking medications continuously. A female patient was concerned if Phenobarbital has an effect on her unborn baby when she was pregnant and others fear if their medications have an interaction with their food and other over the counter medications. There are patients who experience erectile dysfunction, gingival hyperplasia, and allergic reaction. Some patients also reported that they experience bad breath, weight gaining and loss of appetite.
*“My sexual desire was reduced to zero… I hadn't had sexual pleasure since I start taking the medications…” (P1, male)*
*“…Right after I start the medication, I start feeling dizziness like I have been drunk.” (P5, male)*
3. **Concerns about availability and affordability:** Some patient thinks it would cost them so much money because they will take it lifelong. Patients reported that buying generic products from the hospital has reduced their cost of medications but sometimes they encounter unavailability of the medicine form the stock. For this reason, they have to search for their medications in the entire city. Others reported that there is little access to new generation anti-epileptic medications in the country.
  *“…It's going take its own budget…I got scared of having this medication for lifelong because it will have an economic impact in my life.”* (*P7, male*)
4. **Traditional medicine and spiritual healing:** All of the Christian patients said they never used traditional herbal medicine. Some of the Muslim participants for their part mentioned that they have used herbal medicine and they have seen improvement. They couldn't mention the name of the medicinal plant.
*“They give you something compounded with black seed, honey and things I don't know but when compared it to modern medicine it is expensive. I got relieved for some time but it didn't last long.” (P7, male)*
  Almost all Christian patients however used spiritual healing concurrently with their medications or before they started modern medicine. Patients use different types of spiritual healing like praying, fasting and baptizing with holy water. Most patients think using spiritual healing combined with modern medicine have a positive treatment outcome.
*“… I used holy water. I sometimes take the medication with holy water because I should drink it in the morning. I think both are good for my health so I take both.” (P6, female)*

***Relationships with healthcare providers***

Almost all patients complain of being seen by a different physician during their follow up. When a new doctor sees them they are expected to tell their disease history all over again and again. The participants express this as something tedious and challenging. Participants also reported that they don't get enough information on the medication they are taking. They wanted to know if the medication they are taking has serious side effects, drug interaction, and other important information.

*“They didn't tell me about the interaction of Phenytoin with other drugs. I had been taking many medications in the past and I got concern if that has an impact on the level of Phenytoin in my blood.”* (*P8, male*)

***Family support***

All most all of the patients mentioned that they got support from their family. They helped them to remember their pill-taking time and to pass through emotional times. Participants mentioned that instead of worrying about things by oneself it's better to share concerns with family and that helps them to reduce the stress.

*“I got support from my family for example if I had a headache I have to go to a clinic and see a doctor. They treat me better than any child in the family.” (P6, female*)

***Coping procedures***

1. **Delay in treatment seeking:** Participants reported they have tried other alternative medicines. The most common reasons for delay in treatment-seeking behavior were thinking it is caused by an evil spirit and treating with holy water for a long time.
*“For a long time, I was baptized with holy water repeatedly. For that reason, I didn't start modern medicine for two years.” (P9, female)*
2. **Medication-taking behavior:** Study participants also expressed that they did not adhere to the medications. Reasons that they mentioned include increased cost from time to time, being symptom-free for a longer duration and the medication side effects.
*“…mostly I don't adhere to the time. When I take the medications I feel better and start to forget and other times when I get sick I take the medications appropriately for the first week until I get better.” (P2, male)*
*“I stopped the drug in middle because I heard that the medication is not for life long. I thought I was completely cured.” (P10, female)*
3. **Lifestyle coping mechanisms:** Being happy and getting emotionally strong are the two common reported results that participants mentioned in relieving low mood associated with the illness. Participants also mentioned was taking rest and fresh air helped them when they feel they are going to get sick. Having an active religious life is also another coping mechanism mentioned by the participants.
*“For me, making myself free and not stressing about things helps me. Taking rest is also very important.” (P11, female)*

## Discussion

This study is one of the first to explore patients' perceptions towards epilepsy and its management. The findings of this study revealed patient perceptions towards their illness including its cause, timeline, severity and psychosocial consequences and concerns towards their treatment and issues in coping with their treatment. It was apparent that these perceptions and the illness and treatment experiences would negatively affect adherence to medications. On the other hand, there were some social and spiritual factors that seem to positively influence patients' wellbeing and possibly their adherence.

Studies show that there are considerable differences between the perception of patients and doctors towards the illness, which could create barriers to successful clinical management (11). It is important to know about how patients made choice among treatments (12). For example, this study found out when patients become symptom-free for a longer duration they become less compliance towards taking their medication. Some patients discontinue their medication without consulting their physician because they experience side effects. Similarly, it showed that patients who have been seizure free for some time start gradually to reduce their adherence to their medication, as they believe taking it to be pointless (13).

In this study, most of the patients relate epilepsy with possession with evil spirits. Despite patients' level of education, similar response towards the cause of epilepsy was reported among Muslims in Africa and Asia (14). It is a complex interweaving of traditional, folk and spiritually determined condition (15). Similarly, in South Asians, it is believed that epilepsy is caused by spirit possession or attributable to sins committed in the past life (16). Some also consider trauma during childhood and injury as the cause of the disease. They mention trauma as a cause because their doctor asked them if they ever fall during patient assessment. The perception of patients towards the cause epilepsy should be assessed because it determines the health-seeking behavior of patients and therapeutic options they choose.

Even though patients don't communicate with their doctor about depression, some have reported experiences of depression. They, however, don't seem to consider depression as a disease that needs treatment. But, depression has been highly linked with epilepsy with the prevalence of 49.3% among patients with epilepsy (17). This may require more formal consideration by healthcare providers so as to identify and treat those that require it.

In this study, patients experience different side effects based on the medication regimen they are on. Research shows that actual or perceived adverse effects of anti-epileptic medications can increase the likelihood of non- adherence (18). Side effects should be discussed with patients before initiation of treatment and professionals should involve patients during planning treatment regimen because later it can be the reason for patients to discontinue their medication due to unexpected and intolerable side effects.

Patient's perception towards illness affects their behavior towards their treatment choice. A patient who thinks the cause of epilepsy as an evil spirit is most likely to choose holy water as a first resort rather than modern medicine. More than 80% of studies have proven that religion plays an important role in providing mental and physical health (19). Patients perform a religious activity like using holy water and praying alone or in collective to get a cure. In this study, Christian patients tried using holy water before they contact the health care provider. Likewise, research done in HIV positive patients in Ethiopia using a qualitative study method come up with a result of baptizing with holy water and fasting were the two most common reason for patients non-adherent behavior (20).

Participants in this study expressed interest to know more about the cause, and what triggered seizures (21). Furthermore, patients are curious to get more information from their healthcare providers. Trusting relationship with patients and providers can positively affect adherence behavior. Patients think that the medications are necessary but they keep discontinuing taking their medications inappropriately. It's crucial for doctors and other healthcare providers to stress on counseling patients and patients should be convinced about the need for the treatment.

**Limitation of the study:** The interviews were conducted in a hospital environment. This might affect patients' willingness to reveal certain types of information such as health-seeking behavior related to attending traditional healers, and also drug adherence behavior. Patients who are nonadherent or discontinued their medication might not get the chance to participate in this study because it's sampled from the patients who came to the hospital. Despite these limitations, this study was able to present an in-depth exploration of patients' perception towards epilepsy and its treatment with carefully selected patients representing different backgrounds.

In general, it was apparent that patients with epilepsy who were being treated in a hospital setting expressed perceptions about their illness that were different from what is described by the biomedical sciences. This may have contributed to delay in treatment seeking and nonadherence to recommended treatment regimens. Furthermore, the relation that these patients have with their providers is suboptimal which can further contribute to reduced trust in healthcare professionals and the system as a whole which may further contribute to low adherence. This calls for an improvement in the healthcare system with regard to improving chronic care management and the health education provision for patients.
